# Intra-articular remodelling of hamstring tendon grafts after anterior cruciate ligament reconstruction

**DOI:** 10.1007/s00167-013-2634-5

**Published:** 2013-08-27

**Authors:** Rob P. A. Janssen, Sven U. Scheffler

**Affiliations:** 1 Orthopaedic Center Máxima, Máxima Medical Center, Ds. Th. Fliednerstraat 1, 5631 BM Eindhoven, The Netherlands; 2 Chirurgisch Orthopädischer PraxisVerbund (COPV), Breitenbachplatz 8, 14195 Berlin, Germany

**Keywords:** Graft remodelling, ACL, Hamstring tendon, Accelerated rehabilitation, Ligamentization

## Abstract

**Purpose:**

A summary is provided on the existing knowledge about the specific healing phases of the intra-articular hamstring tendon graft used for ACL reconstruction. Differences between human and animal in vivo studies are explained, and implications for the postoperative time period are laid out.

**Methods:**

A systematic review of the existing literature was performed on the topic of tendon remodelling of hamstring grafts in ACL reconstruction using Medline database. Publications between 1982 and 2012 were included. Special focus was directed on in vivo human and animal studies analysing intra-articular free tendon graft remodelling.

**Results:**

Animal and human in vitro and vivo researches have demonstrated three characteristic stages of graft healing after ACL reconstruction: an early graft healing phase with central graft necrosis and hypocellularity and no detectable revascularization of the graft tissue, followed by a phase of proliferation, the time of most intensive remodelling and revascularization and finally, a ligamentization phase with characteristic restructuring of the graft towards the properties of the intact ACL. However, a full restoration of either the biological or biomechanical properties of the intact ACL is not achieved.

**Conclusion:**

Significant knowledge on human cruciate ligament remodelling has been added in the understanding of the processes during the course of graft healing. Most importantly, the remodelling process in humans is prolonged compared to animal studies. While today´s rehabilitation protocols are often extrapolated from findings of animal in vivo healing studies, current findings of human in vivo healing studies might require new post-operative regimens following hamstring ACL reconstruction.

## Introduction

Anterior cruciate ligament (ACL) reconstruction techniques have been improved over the last 10 years, but graft failure is not uncommon: 0.7–10 % [24, 35]. Successful ACL reconstruction requires understanding of several factors: anatomical graft placement, mechanical properties of the selected graft tissue, mechanical behaviour and fixation strength of fixation materials as well as the biological processes that occur during graft remodelling, maturation and incorporation. They influence directly the mechanical properties of the knee joint after ACL reconstruction and, therefore, determine the rehabilitation and time course until normal function of the knee joint can be expected [10, 12, 24, 32–34, 41, 46, 57]. Even though substantial research efforts have been published on various aspects of ACL reconstruction, there is limited knowledge on the biology of the human ACL graft [10, 12, 13, 15, 18, 24, 30, 33, 44–46, 48, 57, 61, 63, 65, 66]. Graft healing after ACL reconstruction occurs at two different sites: intra-tunnel graft incorporation [59, 60] and intra-articular graft remodelling, often referred to as “ligamentization” [4, 10, 24, 30, 33, 34, 44, 46, 53]. This article presents the current knowledge on intra-articular remodelling of ACL grafts with special focus on human hamstring autografts.

## Phases of remodelling

*Animal and human in vitro and vivo research have demonstrated three characteristic stages of graft healing after ACL reconstruction*: an early graft healing phase with central graft necrosis and hypocellularity and no detectable revascularization of the graft tissue, followed by a phase of proliferation, the time of most intensive remodelling and revascularization and finally, a ligamentization phase with characteristic restructuring of the graft towards the properties of the intact ACL [2–4, 24, 27, 33, 36, 43, 65, 66]. However, a full restoration of either the biological or mechanical properties of the intact ACL is not achieved [3, 4, 46].

### Early graft healing phase

This phase is defined as the period from the time of anterior cruciate ligament reconstruction until the fourth post-operative week. It is marked by increasing necrosis, mainly in the centre of the graft and hypocellularity [3–5, 28, 46, 51]. An influx of host cells can be seen into the graft’s periphery between the first and second week [27, 28]. The source of these cells is thought to be the synovial fluid, cells from the stump of the native ACL or bone marrow elements originating from drilling the tunnels. Preservation of the ACL stump and Hoffa fat pad may be beneficial for graft healing in this phase [5, 15, 41]. At the same time, no graft revascularization can be observed [5, 27, 50, 64]. Even though beginning disintegration of collagen fibrils and their orientation can be observed as early as 3 weeks after reconstruction [16], the graft’s overall collagen structure and crimp pattern are maintained [3, 4]. This explains the slow decrease in the mechanical properties of the graft in this early healing phase [40, 46, 50]. During this early healing phase, between 2 and 4 weeks, the lack of sufficient biological graft incorporation is the weak site of the reconstruction with consistent failure by graft pullout [16, 17, 40, 58], therefore requiring and relying on appropriate mechanical graft fixation. A shift towards the intra-articular graft region becoming the weak link is noted during the proliferation healing phase when the maximum remodelling activity seems to interfere with the mechanical strength of the healing graft [16, 40, 61].

### Proliferation phase of graft healing

The proliferation phase is defined as the period between 4 and 12 weeks after ACL reconstruction.

This phase is characterized by maximum cellular activity and changes of the extra-cellular matrix, which are paralleled by the lowest mechanical properties of the reconstructed ACL graft. Graft necrosis leads to a release of growth factors, which stimulate cell migration and proliferation as well as extracellular matrix synthesis and revascularization [22, 26, 29, 51, 64]. An increased number of specific fibroblasts so-called myofibroblasts appear. They are responsible for the restoration of the in situ tension that is required for the later ligamentization phase [36, 46, 56, 62]. At the end of the proliferation phase, cell density is still increased, but recedes towards the intact ACL’s cellularity [6, 21, 24, 46, 51, 55, 58]. Revascularization of the graft starts from the fourth post-operative week [5, 46, 55, 61], progressing from the periphery of the graft to the entire graft diameter at 12 weeks [42, 55].

Animal studies have shown that the mechanical properties of the graft are at its weakest at 6–8 weeks. Three factors contribute to the decline in the grafts’ mechanical properties: (a) increased revascularization and extra-cellular infiltration, (b) loss of regular collagen orientation and crimp pattern and (c) decrease in collagen fibril density, followed by increased collagen synthesis with a shift from large-diameter collagen fibrils to small-diameter fibrils [6, 9, 16, 20, 21, 27, 46, 51, 52, 54, 58, 60, 61]. Furthermore, increased collagen III synthesis (with lower mechanical strength than type I collagen) may further explain why a full restoration of the mechanical strength of the intact ACL has not been observed in any in vivo model even after 2 years of healing [32, 42, 46, 52].

The reduced mechanical properties of healing grafts in animal models seem to contradict the successful clinical outcomes after ACL reconstruction with immediate aggressive rehabilitation in humans. Significant differences were found in biopsy studies between the remodelling activity of human ACL grafts during the first 3 months and the healing graft in animal models. The complete loss and replacement of all intrinsic graft have not been observed in human biopsy studies [25, 43]. The excessive graft necrosis in animals could not be confirmed in humans, where necrosis or degeneration never involved in more than 30 % of the graft’s biopsies [25, 35, 43]. Neovascularization was not as excessive in humans [25]. Large areas of human healing graft stay unchanged displaying tendinous structure with normal collagen alignment and crimp pattern [25]. Loss of collagen organization was only detected in areas of neovascularization in human biopsies, which corresponds to the findings in animal studies [24, 46]. However, human biopsy studies confirm the remodelling cascade of (limited) graft necrosis, recellularization, revascularization and changes in collagen crimp and composition during the early healing and proliferation phases, suggesting that also the human ACL graft might have its lowest mechanical strength around 6–8 weeks post-operatively [43, 65]. Loading of the graft must be high enough to stimulate graft cells to produce cellular and extra-cellular components for preservation of graft stability, but without compromising graft integrity, which might result into an early stretch-out of the ACL reconstruction [46].

### Ligamentization phase of graft healing

The ligamentization phase involves the continuous remodelling of the healing graft towards the morphology and mechanical strength of the intact ACL from 12 weeks onwards. A clear endpoint is not known for certain changes still occurring even years after reconstruction. In animal models, cellularity slowly returns to values of the intact ACL between 3 and 6 months post-operatively [42, 46, 55, 61]. Vascularity throughout the graft decreases and returns to values of the intact ACL between 6 and 12 months, when vessels become evenly distributed throughout the entire graft [5, 46, 55, 61]. Collagen fibres regain their organization, which microscopically resembles the appearance of the intact ACL around 6 and 12 months after reconstruction [46, 62]. However, the initial loss in collagen crimp and strict parallel alignment of the proliferation phase is only partially restored [46, 62]. The heterogeneous composition of collagen fibres of varying diameter of the intact ACL is never restored [1, 21, 31, 58]. It has been shown that the mechanical properties of the ACL-reconstructed knee joint improve substantially during the phase of ligamentization and reach their maximum properties at around 1 year. However, not a single animal study has demonstrated that the structural properties (e.g. failure load, stiffness) of the healing graft could surpass 50–60 % of the intact ACL [6, 9, 16, 21, 37, 38, 40, 46, 60, 61]. While human biopsy studies showed substantial differences from animal models during the proliferation phase, the ligamentization phase is rather similar in both models in terms of biological progression. However, the timeline of these biological changes is different: studies in humans have shown a prolonged remodelling process compared to animal models [10, 12, 24, 30, 33, 43, 44, 46, 53, 65, 66].

## Remodelling of human hamstring autografts after ACL reconstruction

When interpreting animal data with regard to changes occurring in human autografts, important clinical factors such as graft isometricity, anatomical positioning, patient compliance, healing response, vascularity, biomechanical strength and post-operative rehabilitation must be considered. These factors are difficult to control in animal models. Nevertheless, the results of animal studies are important, because human research has been limited to post-mortem and second-look arthroscopic evaluation [33]. Research on remodelling of human hamstring autografts after ACL reconstruction can be divided into MRI studies and biopsy studies [10, 12, 13, 15, 24, 30, 33, 44, 47, 57, 66]. The current knowledge on remodelling of human hamstring ACL grafts and rehabilitation will be presented in the next sections.

### MRI studies of human hamstring ACL grafts

MRI studies have examined the revascularization of human hamstring autografts after ACL reconstruction [13, 15, 18, 57]. In a gadolinium-enhanced MRI study, Howell et al. [18] did not demonstrate any discernible blood supply in an unimpinged 4-strand hamstring ACL graft during the 2 years of implantation. The graft retained the same hypovascular appearance as the normal posterior cruciate ligament. In contrast, the periligamentous soft tissues were richly vascularized and covered the graft by 1 month. They postulated that the viability of an unimpinged, human hamstring ACL graft may depend more on synovial diffusion than on revascularization. This is in contrast to findings in animal studies, where gadolinium-enhanced MRI showed significant upregulated neovascularization during the first 3 post-operative months [61]. This underlines the differences in remodelling between humans and animal models. Although human biopsy studies have shown that neovascularization of the hamstring graft occurs, the extent of vascularity in humans might be below the threshold detectable with gadolinium-enhanced MRI [46]. Gohil et al. [15] investigated the effect of minimal debridement of the stump of the ruptured ACL on revascularization of 4-strand human hamstring ACL autografts. They concluded that minimal debridement led to earlier revascularization within the midsubstance of the ACL graft at 2 months, but found no evidence that the minimal debridement accelerated the recovery of graft strength. Other authors examined the effect of autologous platelet concentrate on remodelling of 4-strand human hamstring ACL autografts with a standardized accelerated rehabilitation protocol. Vogrin et al. [57] used contrast-enhanced MRI and found that revascularization of the graft only started at 4–6 weeks after ACL reconstruction. Autologous platelet concentrate did not influence intra-articular remodelling of hamstring grafts [13, 57]. The revascularization of the human hamstring graft at 4–6 weeks correlates with the proliferation phase of graft healing.

### Biopsy studies of human hamstring ACL grafts

Human biopsy studies have examined the remodelling process of the hamstring tendon autograft at various time intervals after clinically successful ACL reconstruction [10, 12, 13, 24, 30, 33, 44, 47, 66]. The human hamstring autograft remains viable after reconstruction and shows typical stages of remodelling: early phase graft healing, a proliferation phase and a ligamentization phase [10, 12, 24, 44]. Graft integrity is much less compromised during the early healing and proliferation phase in human ACL grafts, which might allow for the assumption that the mechanical properties are also substantially higher than in animal models during the first 3 post-operative months [10, 25, 46].

**Fig. 1:**
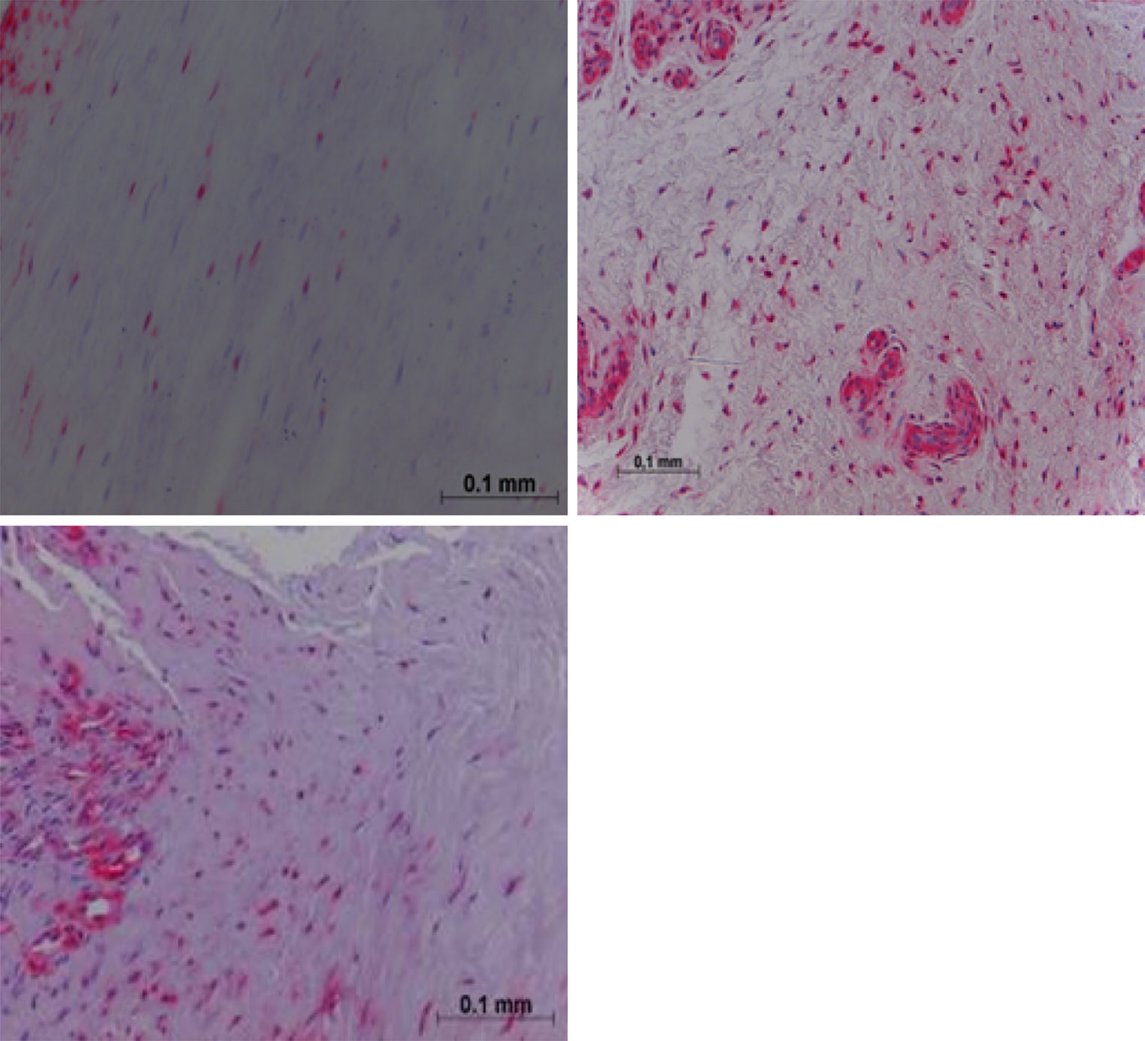
Alpha-smooth staining biopsies of human hamstring ACL graft showing a moderate number of myofibroblasts 6–12 months (*top left*) compared to 13–24 months (*top right*) and over 24 months (*bottom left*) after ACL reconstruction. Note an increased number of myofibroblasts and vessels in biopsies at 13–24 months and over 24 months after ACL reconstruction (reproduced with permission from [24])

Focus of human hamstring biopsy studies has been the proliferation and ligamentization phases of graft healing, as most biopsies were taken at second-look arthroscopies from 4 months onwards after ACL reconstruction. Janssen et al. [24] examined 67 patients who underwent retrieval of midsubstance biopsies after clinically successful 4-strand hamstring autograft ACL reconstruction with a standardized accelerated rehabilitation programme. Cellular density and vascular density were increased up to 24 months after ACL reconstruction. Especially the strong increase in myofibroblast density, from 13 up to 24 months, indicated an active remodelling process from 1 to 2 years (Fig. 1). Furthermore, vessel density increased over 24 months, whereas cell and myofibroblast density decreased but stayed higher than native hamstring and ACL controls. Collagen orientation did not return to normal in the study period (up to 117 months after ACL reconstruction).

Human biopsy studies that analysed changes of the extracellular matrix observed changes that are in line with the findings of animal models. Marumo et al. [33] found that the collagen cross-links of hamstring tendon autografts had changed from time zero, when they were significantly different from the intact ACL, to 1 year post-operatively, when both grafts had acquired cross-link ratios that were identical to the intact ACL, confirming the ligamentization process found in animal models. Interestingly, biopsy specimens taken at 6 months still showed significantly different cross-link ratios of the healing grafts compared to the intact ACL, which is different from the earlier cross-link restoration found in animal models [30, 46]. This also confirms the different timeline of the remodelling of human ACL grafts. Zaffagnini et al. [66] confirmed the observations in animal models [22, 31, 61] that human hamstring ACL grafts showed a replacement of large- by small-diameter fibrils, which did not change even after more than 2 years. Sanchez et al. [44] showed that use of platelet-rich plasma preparation rich in growth factors (PRGF) in hamstring ACL autografts resulted in temporal histological changes during the 6- to 24-month post-operative period in comparison with non-PRGF-treated grafts. Biopsies were taken from the periphery of the hamstring autograft, and the authors question whether these ACL substitutes entirely replicate the full mechanical properties of the intact ACL. A better understanding of the graft biology in human ACL reconstruction will depend on the possibility to obtain core biopsy samples of the grafts [10].

In summary, human hamstring ACL autografts undergo a process of adaptation rather than full restoration of the intact ACL’s biological properties, which takes at least 1 year after reconstruction.

## Human hamstring remodelling and rehabilitation

Knowledge about the duration of the remodelling process of ACL grafts may influence and improve rehabilitation protocols [24, 33, 46]. Arthroscopic findings and clinical results after hamstring ACL reconstruction are found to be satisfactory with both accelerated and less aggressive rehabilitation programs [7, 8, 19, 23, 24, 33]. Advantages of accelerated rehabilitation protocols after ACL reconstruction are earlier normal function of the knee [8, 19, 49] and have ability to return to even most strenuous activities after primary ACL reconstruction at 6 months [46]. However, some authors found that early return to vigorous physical activity may increase the risk of greater knee laxity after ACL reconstruction [14, 35]. Biological findings have shown that human hamstring ACL graft remodelling takes at least 1 year after ACL reconstruction and is prolonged compared to animal models, on which current rehabilitation protocols are based after ACL reconstruction [11, 12, 24, 30, 33, 44, 46, 47, 55, 56, 58–62, 66]. Based on these findings in their biopsy study, Janssen et al. [24] question whether accelerated rehabilitation is to be recommended after 4-strand hamstring ACL reconstruction. It is agreed that ACL graft healing can only progress if mechanical loading occurs; however, the most adequate magnitude at the varying phases of healing is still not clarified [35, 39, 46, 54]. It is crucial to understand what rehabilitation activities might lead to excessive ACL tensioning and therefore must be avoided during the first 3 post-operative months.

No final conclusions can be drawn on the mechanical strength of healing ACL grafts in humans with no available techniques for in vivo measurement of their mechanical properties. Even though it is not fully understood what the exact mechanisms are that guide the remodelling process, it seems to be important that physiological knee joint mechanics are restored to provide the same mechanical stimulus to the healing ACL graft as to the intact ACL. This guides adequate remodelling that will maintain initial graft integrity and (partial) cell viability, while initiating cellular and extra-cellular proliferation and differentiation to adapt the graft to its new biological and mechanical environment.

## Conclusion

Hamstring tendon grafts remain viable after ACL reconstruction. The graft undergoes 3 characteristic stages of graft healing after ACL reconstruction: an early graft healing phase with limited graft necrosis and hypocellularity and no detectable revascularization of the graft tissue, followed by a phase of proliferation, the time of most intensive remodelling and revascularization and finally, a ligamentization phase with characteristic restructuring of the graft towards the properties of the intact ACL. An adaptation of the healing graft towards the intact ACL occurs without a full restoration of either the biological or mechanical properties of the intact ACL. Future research will have to be directed to (a) optimizing cruciate ligament reconstructions to fully restore the anatomy and function while providing the mechanical strength of the intact cruciate ligaments, (b) developing biological treatment options that impact on graft healing especially during the early and proliferation phase to optimize extra-cellular matrix remodelling and avoid excessive remodelling activity that might impair mechanical integrity of the healing graft and (c) to better differentiate the “good” from the “bad” remodelling changes, so that the time to return to full activity without any restrictions can be reduced.
